# Two-color immunocytochemistry for evaluation of effusion fluids for metastatic adenocarcinoma

**DOI:** 10.4103/1742-6413.59887

**Published:** 2010-02-10

**Authors:** Vinod B Shidham, George Varsegi, Krista D'Amore

**Affiliations:** Department of Pathology, Medical College of Wisconsin, Milwaukee, Wisconsin, USA

**Keywords:** Serous effusion, immunohistochemistry, immunocytochemistry, two-colour immunostaining, metastasis, serous fluid, serous cavity, dual immunostaining

## Abstract

**Background:**

The evaluation of serous fluids by conventional one color immunocytochemistry is complex and challenging.

**Design:**

We selected and studied 37 serous fluid cytology specimens (23 pleural, 13 peritoneal, 1 pericardial), collected over a 4-year period. They were unequivocally positive for metastatic adenocarcinoma based on clinical correlation, cytomorphology, and one color immunocytochemistry on cell block sections. 3 μm serial sections of cell blocks were immunostained by a two chromogen method (peroxidase with brown chromogen followed by alkaline phosphatase with red chromogen). Combinations evaluated were: A- vimentin followed by cytokeratin (CK) 7; B- calretinin followed by BerEP4, C- calretinin followed by CK 20. Additionally, difficulty of interpretation was evaluated on a scale of 1(easy) to 5 (difficult). Cases demonstrating decreased or complete loss of immunoreactivity with alkaline phosphatase red chromogen system were also evaluated with routine one color immunostaining by alkaline phosphatase and peroxidase individually. The pretreatments for antigen retrieval and antibody dilutions were identical to those used for conventional one color immunostaining with respective immunomarker.

**Result:**

Combination ‘A’ showed correlation with the immunoreactivity pattern observed with one color immunostaining. However, the immunoreactivity of the second immunomarker was compromised in combinations B and C. In the latter group, the sections immunostained with one color alkaline phosphatase indicator system also showed weak immunoreactivity or complete loss of immunoreactivity for the corresponding second immunomarker. However, the peroxidase system showed proper immunoreactivity for those immunomarkers. Average difficulty of interpretation for the two color method was 1.06 (range- 1 to 2) as compared to 2.95 (range: 1 to 5) with the one color method. This difference was statistically significant (two-tailed *P*<.0001, paired t test). The higher scores of difficulty were observed in cases with a paucity of tumor cells and cases with predominance of isolated tumor cells.

**Conclusion:**

Dual immunostaining facilitated identification of the foreign population of malignant cells in effusion fluids with objective, reproducible precision. However, due to relatively lower sensitivity of alkaline phosphatase as a second indicator system, immunoreactivity was diminished for BerEP4 and lost for CK 20.

## INTRODUCTION

Evaluation of effusion cytology is one of the most challenging areas in diagnostic cytopathology. A remarkably wide cytomorphological spectrum of reactive mesothelial cells overlaps with various benign and malignant processes.[1–5] Due to these limitations, a significant proportion of effusion fluids are difficult to interpret with objective certainty by cytomorphology alone, especially in cases with scant tumor cells particularly in metastatic disease from well differentiated mammary and ovarian carcinoma. The difficulty of this challenge, however, may vary from institution to institution. This may depend on multiple factors including the level of training or experience of the interpreter, patient demographics (such as type of prevalent diseases, predominant sex and age groups), and the quality of technical support for cytopreparatory processing. Proper application of immunocytochemistry is a valuable ancillary tool, introducing a component of objectivity to this exercise.[6–9]

A simple approach for confirming metastatic disease in effusions is detection of a ‘second-foreign’ non-inflammatory, nonmesothelial population of cells.[5] In addition to cytomorphologic evaluation, immunocytochemistry is commonly applied as a relatively objective tool for this purpose. An immunopanel highlighting the mesothelial and inflammatory cells contribute to the understanding of the basic topography of different components in the effusion specimen. This strategy facilitates straightforward detection and confirmation of a ‘second-foreign’ population by the ‘Subtractive Coordinate Immunoreactivity Pattern’ (SCIP) approach.[6] ‘Subtracting’ the immunoprofiles of various basic components of effusion specimen (reactive mesothelial cells and inflammatory cells), highlights the existence of a ‘second foreign population’ if present. ‘Second foreign population’ in effusion is usually consistent with metastatic disease. As described in detail by Shidham and Atkinson, this scheme to localize, identify, and characterize the ‘second foreign population’ in cell-block sections by immunocytochemistry is referred to as the SCIP approach.[6]

For proper application of the SCIP approach, it is important to evaluate the same cells when constructing coordinate immunoreactivity pattern for various immunomarkers. Since the same cells cannot be followed on different cytology smears, they are not ideally suited for routine immunocytochemical evaluation of effusion fluids.[6] Serial sections of cell-blocks show the same cells in adjacent levels which allow proper evaluation of the coordinate immunoreactivity pattern.[10,11] Application of the SCIP approach facilitates careful evaluation of this coordinate immunoreactivity for detection of a *second-foreign'* population of cells especially in challenging cases.[6,12]

However, even with the SCIP approach, the *‘second-foreign’* population of neoplastic cells in conventionally immunostained sections using single chromogen may be difficult to interpret in multiple sections on different slides. This is exceptionally challenging when the neoplastic cells are scant or are dispersed as solitary cells. Dual color immunochemistry with reciprocally complimentary combinations of different immunomarkers in concert with SCIP approach may simplify the process with additional benefits of using fewer sections. In this study, we examined dual color immunocytochemistry by utilizing three pairs of different immunomarkers to evaluate effusion fluids for metastatic adenocarcinoma.[13]

## MATERIALS AND METHODS

The current retrospective study was performed after approval by the Institutional Review Board (IRB) on 37 serous fluid cytology specimens (23 pleural, 13 peritoneal, 1 pericardial, over a period of 4 years) interpreted as positive for metastatic malignant cells. The initial diagnoses were made with the help of cytomorphology, one color immunocytochemistry on cell block sections, and clinical history.

**Figure 1:**
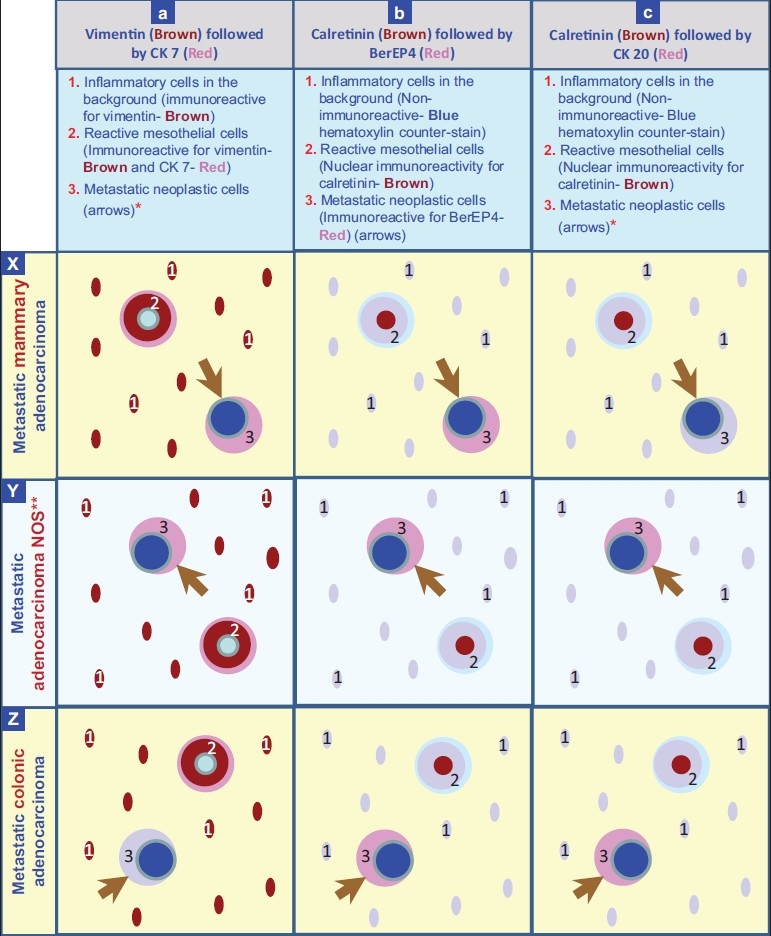
Staining pattern with dual color immunostaining counterstained with hematoxylin. *The neoplastic cells are immunoreactive for CK 7 (Red) in metastatic mammary carcinoma (a×) and immunoreactive for CK 20 (Red) in metaplastaic colonic adenocarcinoma (cZ). **The example shown in Y is a metastatic adenocarcinoma with concordant immunoreactivity for CK 7 and CK 20.

The cell blocks were prepared by HistoGel™ method.[14,15] 3 μm serial sections of cell blocks were immunostained by a dual chromogen method (peroxidase with brown chromogen followed by alkaline phosphatase with red chromogen) [Table 1]. Combinations evaluated for confirming second population of neoplastic cells are as follows [Table 2, Figure 1]:

**Table 1:** Summary of protocol for dual color immunostaining

Deparaffinize the section with xylene Clear xylene with a graded series of ethanol Methanol: 10 dips 50% H_2_O_2_ in Methanol- 12 minutes Deionized water: 10 dips Pretreatment (see Table 2 for details) Rinse with Tris buffer (pH 7.6). First primary antibody (300 μl) (For dilutions and other details see Table 2)- 30 minutes Rinse with Tris buffer (pH 7.6). Peroxidase labeled polymer reagent (300 μl)- 30 minutes [(Envision+ System- HRP Labeled Polymer) (Dako Code K4000 and 4001)] Rinse with Tris buffer (pH 7.6). Brown chromogen (300 μl)- 7 minutes [3,3' diaminobenzidine HCL (Dako K3467)] Rinse with deionized water. 0.1 HCl (300 μl) (To wash away excess polymer)- 5 minutes Rinse with Tris buffer (pH 7.6). Second primary antibody (300 μl) (For dilutions and other details see Table 2)- 30 minutes Alkaline phosphatase labeled polymer reagent (300 μl)- 30 minutes [Envision System- AP (Dako Code K4017 and 4018)] Rinse with Tris buffer (pH 7.6). Red chromogen (300 μl)- 14 minutes [Liquid permanent red (Dako K0640)] Rinse with deionized water. Counterstain 30 seconds with light green diluted 1:5 with deionized water Rinse with deionized water. Dehydrate quickly in100% alcohol* Clear quickly with 10 dips in Xylene* Coverslip with permount

Although not required and not followed in this protocol, a protein block may be added as indicated by using ‘Dako Protein Block Serum-Free Ready-to-use (Code XO909) after step #7 and before step# 8,

* The final red colored product of red chromogen may be lost if left in alcohol and xylene for longer duration

**Table 2:** Antibodies used during initial evaluation

*Immunomarker*	*Antibody details*	*Dilution*	*Pretreatment*	*First or second*	*Final color*
Vimentin (in A)	Monoclonal, clone Vim 3B4, Dako	1:200	Proteinase K** Code- S3020 5 minutes	First in combination A	Brown [Cytoplasmic]
Cytokeratin 7 (in A)	Monoclonal, clone- OV-TL 12/30 Dako	1:400	Proteinase K** Code- S3020 5 minutes	Second in combination A	Red [Cytoplasmic]
Calretinin (in B and C)	Monoclonal, Clone- Dak Calret 1 Dako	1:100	Heat induced epitope retrieval (HIER)*	First in combination B and C	Brown [Nuclear (and cytoplasmic)]
BerEP4 (in B)	Monoclonal, Clone- BER-EP4 Dako	1:100	Heat induced epitope retrieval (HIER)*	Second in combination B	Red [Cytoplasmic]
Cytokeratin 20 (in C)	Monoclonal, clone- Ks 20.8 Dako	1:100	Proteinase K** Code- S3020 5 minutes	Second in combination C	Red [Cytoplasmic]

* HIER, Dako Cytomation (Dako, Code S1699) Target Retrieval Working Solution for 35 minutes at 99°C, followed by cooling for 20 minutes at room temperature.

** Proteinase K, Ready-to-use (Dako, Code- S3020) was diluted 1:1 with Tris-HCl buffer (pH 7.6), Antibodies were diluted with Dako Antibody Diluent (Dako, Code- SO809)

Dako: Dako Denmark A/S, Produktionsvej 42, DK-2600 Glostrup, Denmark

Combination A. First vimentin (brown) followed by cytokeratin 7 (red), Combination B. First calretinin (brown) followed by BerEP4 (red), Combination C. First calretinin (brown) followed by cytokeratin 20 (red)

A: vimentin (brown) followed by cytokeratin (CK) 7 (red),

B: calretinin (brown) followed by BerEP4 (red), and

C: calretinin (brown) followed by CK20 (red).

The combination of immunomarkers in each pair, and sequence of each immunomarker in corresponding pairs, were decided according to the method of pretreatment (antigen retrieval step) suitable for different immunomarkers and their immunospecificity for various components of the effusion specimen.

Cases with negative or decreased immunoreactivity for the second immunomarker within a combination were also subsequently re-evaluated with conventional one color immunostaining with alkaline phosphatase and peroxidase indicator systems individually. The pretreatments for antigen retrieval and antibody dilutions were similar to those used for conventional one color immunostaining for all immunomarkers [Table 2]. One senior and one junior pathologist reviewed the slides on double headed microscope after initial discussion about how to interpret with SCIP approach. The ease or difficulty of interpretation was decided based on subjective consensus by both the interpreting pathologist depending on the time required to reach the final decision. The scale of difficulty from 1 to 5 was also statistically analyzed for comparative evaluation [Table 3]. As this was related to coming to the final conclusion not the diagnosis, the cases which did not demonstrate proper imminostaining of second color were just interpreted as negative with ease, but the results were wrong as mentioned in Table 4.

**Table 3:** Level of difficulty of interpretation with one color versus two color immunostaining

Case no. (All case numbers were not used)	Level of difficulty	

	1 color	2 color
1	3	1
2	4	1
3	5	1
4	3	1
5	3	2
7	1	1
8	4	2
10	5	1
11	5	1
12	3	1
13	5	1
14	1	1
15	3	1
16	3	1
17	3	1
18	5	1
20	5	1
21	4	2
22	3	1
23	3	1
24	1	1
25	3	1
26	1	1
27	3	1
28	3	1
30	1	1
31	2	1
32	1	1
33	5	1
34	5	1
35	1	1
36	1	1
38	1	1
39	5	1
41	1	1
42	2	1
43	3	1
Range	1 to 5	1 to 2
Mean	2.95*	1.06*

* Two-tailed P value < .0001, paired t test. (1 = easy, 5 = difficult), Number 9, 19, 29, 37, 40 were not used.

**Table 4:** Correlation of two color immunostaining results with one color immunostaining

*Primary neoplasm*	*Total*	*CK7*			*BerEP4*			*CK20*		
				
		*1c CK7*	*2c V/CK7*	%*	*1c BerEP4*	*2c Cal/BerEP4*	%*	*1c CK20*	*2c Cal/CK20*	%*
Breast	13	13	13	100	13	7	54	0	0	×
Gastrointestinal	7	7	7	100	7	5	71	7	0	0
Pancreas	1	1	1	100	1	1	100	0	0	×
Lung	8	8	8	100	8	8	100	0	0	×
Ovary	4	4	4	100	4	3	75	0	0	×
Peritoneal	1	1	1	100	1	0	0	0	0	×
Gynecologic	3	3	3	100	3	2	67	0	0	×

2c = two color; 1c = one color; V = vimentin; Cal = calretinin; CK = cytokeratin.

* cases in which immunostaining combination was diagnostic.

## RESULTS

Dual color immunochemistry simplified unequivocal interpretation of effusions for metastatic carcinoma [Table 4].

**Figure 2:**
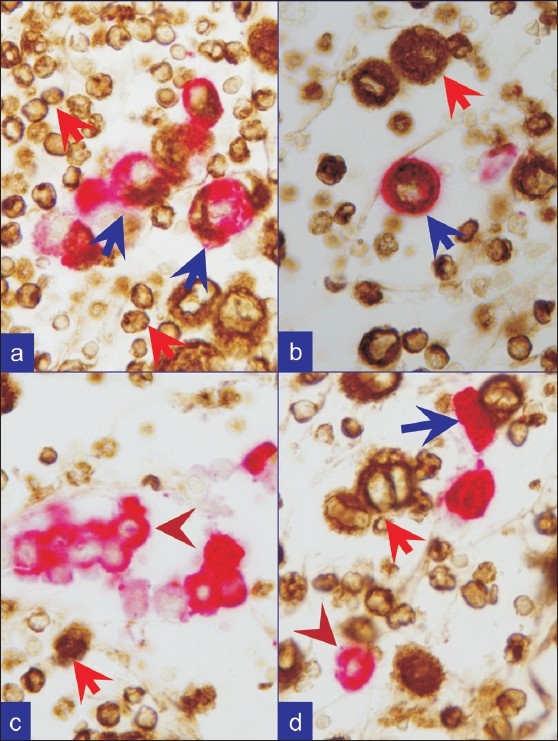
Metastatic breast carcinoma. a, b, c, and d. Combination A: First vimentin (brown) followed by Cytokeratin 7 (red). Reactive mesothelial cells (blue arrows) are brown (vimentin) and red, (CK-7). Inflammatory cells (red arrows) are brown (vimentin). Metastatic neoplastic cells (brown arrowheads) are only red with cytokeratin 7 immunoreactivity alone, consistent with breast as primary site

In combination ‘A’ [Figure 1 and 2], the reactive mesothelial cells showed brown cytoplasmic immunostaining for vimentin, and red cytoplasmic immunostaining for CK 7. The inflammatory cells in the background were brown (cytoplasmic immunostaining for vimentin). If a CK 7 immunoreactive second population was present, only red cytoplasmic immunoreactivity was visible. If a CK 7 non-immunoreactive second population was present, it was seen as non-immunostained cells exhibiting only hematoxylin counter-staining.

**Figure 3:**
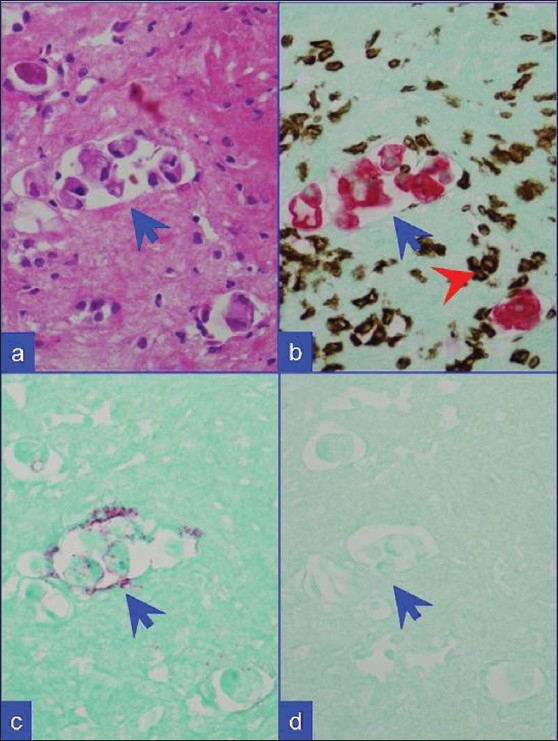
Metastatic breast carcinoma. (a) HE stained cell block section. (b) Combination A: Metastatic mammary carcinoma cells (blue arrows) with red CK 7 immunoreactivity. Inflammatory cells with brown vimentin immunoreactivity (red arrowhead) in the background. (c) Combination B: The neoplastic cells show weak BerEP4 immunoreactivity (red). (d) Combination C: They are non-immunoreactive for CK 20. ‘c’ and ‘d’ specimen did not show significant number of mesothelial cells with lack of brown nuclear immunoreactivity for calretinin in this field as in ‘b’ (red-CK-7 with brown- vimentin)

**Figure 4:**
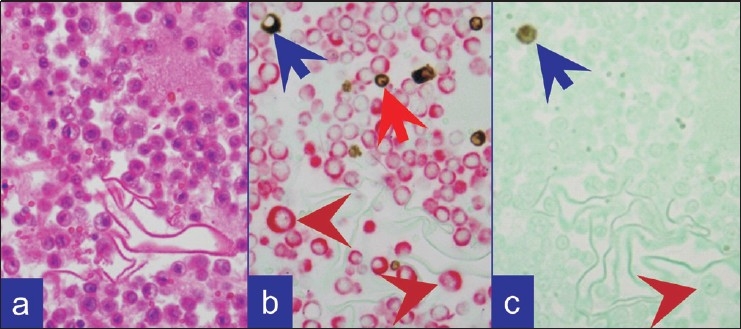
Metastatic adenocarcinoma as single cells. (a) HE stained cell block section. (b) Combination A: Predominantly isolated metastatic neoplastic cells (brown arrowheads) with CK 7 immunoreactivity (red). A few vimentin immunoreactive (brown) inflammatory cells (red arrow) and occasional reactive mesothelial cell (blue arrow) in the background. (c) Combination B: Occasional calretinin immunoreactive (brown) mesothelial cell (blue arrow). Isolated metastatic neoplastic cells in the background (brown arrowhead) did not show red color for BerEP4 (immunoreactive with single color peroxidase method)

In combination ‘B’,[Figure 3, 4] the reactive mesothelial cells showed brown nuclear (and cytoplasmic) immunostaining for calretinin with red cytoplasmic immunostaining for BerEP4. The inflammatory cells in the background were present as non-immunostained cells, stained by the counterstain only. The BerEP4 immunoreactive second population showed red cytoplasmic immunoreactivity. However, BerEp4 immunoreactivity with expected red immunostaining was either diminished in most of the cases [Figure 3c] or lost in some cases [Figure 4c].

In combination ‘C’, [Figure 3, 4] the reactive mesothelial cells showed brown nuclear (and cytoplasmic) immunostaining for calretinin. If CK20 positive neoplastic cells were present, they would demonstrate red cytoplasmic immunostaining only. However, the expected red immunostaining for CK20 was not observed in any case. CK20 immunoreactive metastatic carcinoma cells were present in 7 cases with metastatic gastrointestinal carcinoma with one color immunohistochemistry by the peroxidase method. The inflammatory cells in the background were present as non-immunostained cells stained by the counter-stain only. A ‘second population’ of metastatic carcinoma cells which were negative for CK20 (but may have been immunoreactive for CK7) were also seen as non-immunostained cells stained by the counter-stain only [Figure 3d].

The immunoreactivity pattern observed in Combination ‘A’ showed good correlation with conventional one color immunostaining in all 37 cases {Breast (13), Gastrointestinal (7), Pancreas (1), Lung (8), Ovary (4), Peritoneal (1), and Gynecologic (3)} [Table 4]. The original immunoreactivity pattern of the second immunomarker was compromised in combinations ‘B’ and ‘C’. In sections immunostained by the dual color method, BerEP4 showed decreased intensity in 37 cases, with complete negativity in 11 cases. CK20 showed complete loss of immunoreactivity in all 7 cases of metastatic gastrointestinal adenocarcinomas. In these cases, subsequent immunostaining with conventional one color method using peroxidase indicator system showed strong immunoreactivity for BerEP4 in all 37 cases in group B and for CK20 in all seven cases in group C. Repetition of one color immunostaining with alkaline phosphatase as the indicator system in these cases showed corresponding weak immunostaining or complete loss of immunoreactivity for BerEP4 and CK20, with the same antibody dilution used for the peroxidase indicator system.

On a scale of 1 to 5, the average difficulty of interpretation with the single color method was 2.95 (range 1 to 5). However, interpretation with the dual color method was significantly easier with an average difficulty score of only 1.06 (range 1 to 2). This difference was statistically significant (two-tailed *P* <.0001, paired *t* test) [Table 3]. Higher scores of difficulty were observed in cases with scant tumor cells or predominantly single tumor cells, especially with the conventional one color immunocytochemistry method. The interpretation of some specimens with the one color immunocytochemistry method was impossible if the sections immunostained were not oriented identically on all slides or if the serial relationship was not properly identified on individual slides for proper SCIP approach.[6,12]

## DISCUSSION

Two positive and two negative mesothelial markers may be sufficient for distinguishing mesothelial cells and adenocarcinoma cells in tissue specimens. However, this challenge is more complex and requires a special approach for effusion specimens. Cytology smears generally are not suitable for immunocytochemical evaluation of effusions and so cell block sections are required.[6,11,16] The proper evaluation begins with appropriate methodology for cell block preparation to get optimum diagnostic cells in cell block sections.[14,15,16] Although subtle, the significant assistance provided by the architectural relationship of different components in surgical pathology specimens is absent in cell block sections of effusion fluids. This intricacy is further enhanced by the significant cytomorphological overlap between reactive mesothelial cells and malignant cells, especially in low grade malignancies from breast and ovary.

This necessitates the strategy of creating a basic map of different components in cell block sections of effusion specimens for topographic understanding. By ‘subtracting’ immunoprofiles of various basic components of the specimen, the presence of a ‘second foreign population’, consistent with metastatic disease can be identified. This scheme to localize, identify, and characterize the ‘second foreign population’ in cell-block sections by immunocytochemistry is referred to as the ‘*Subtractive Coordinate Immunoreactivity Pattern*’ (SCIP) approach.[6] However, application of SCIP approach with conventional one color immunocytochemistry may not easily be applied to all effusions, especially cases with rare metastatic cells of well differentiated ovarian and mammary carcinoma and cases with isolated cells.

Dual color immunostaining with the SCIP approach identified the foreign population of malignant cells [Table 4, Figure 1] with significant ease [Table 3]. This method was most effective in combination ‘A’ (vimentin and CK7). However, immunoreactivity for BerEP4 (in combination ‘B’) was diminished and for CK20 (in combination ‘C’) was completely lost. The reason for this interference, after initial evaluation, appears to be related to the lower sensitivity of alkaline phosphatase as the indicator system when used as second color in dual color combination. Although, not evaluated for this study, increased concentration of antibodies and/or modifying the antigen retrieval method may help.

Dual color SCIP approach, utilizing combination ‘A’ with brown vimentin immunostaining and red CK 7 immunostaining [Figure 2], was easier to evaluate when compared to the results from evaluation by conventional one color immunostaining. Information from combination ‘B’ provided additional confirmation by offering more immunoprofile data on BerEP4 immunoreactivity of the same cells (consistent with adenocarcinoma) in many cases. In combination ‘A’, reactive mesothelial cells were brown or brown and red (vimentin and CK 7 immunoreactivity) and inflammatory cells were brown (vimentin immunoreactive) [Figure 2a,b]. Any cells immunostaining exclusively red (CK 7 immunoreactivity only) [Figures 2c,d; 3b; 4b] or not immunostained at all may be considered consistent with metastatic disease. Depending on the primary site these cells were red (breast, ovary, lung etc) or unstained (other carcinomas including colonic carcinoma and other tumors) [Figure 1]. With combination B, if the metastatic disease was adenocarcinoma, the cells were usually seen as red immunostaining (BerEP4 immunoreactivity) [Figure 3c] amongst variable numbers of reactive mesothelial cells with brown nuclei (calretinin immunoreactivity).

Immunostaining protocols for more than two colors are also described.[17] Based on the reported literature, a variety of diagnostic combinations may be evaluated. As identified during our study, a protocol related interference in immunoreactivity patterns is a distinct possiblity with confounding consequence. This underscores the significance of standardization and evaluation of any combination and related immunostaining protocols prior to clinical application. In addition, the contrast of colors used is important. The protocol for brown and red colors used in this study showed good contrast [Figure 2–4]. Recently we observed excellent contrast with the dual staining protocol performed on some of the cases using ‘ultraView™ Universal DAB Detection Kit (with Brown chromogen)’ followed by ‘ultraView™ Universal Alkaline Phosphatase Red Detection Kit (with Red chromogen)’ on BenchMark^®^ ULTRA Flexible Automation Continuous Access Slide Preparation System.[18]

In summary, dual staining facilitated identification of the foreign population of malignant cells in serous fluids with objective precision in fewer sections. It increased the ease of interpretation as compared to the routine single color immunostaining approach. However, immunoreactivity for BerEP4 was diminished and for CK20 was lost, due to the relatively less sensitive nature of the second immunostaining indicator system (alkaline phosphatase). Higher concentrations of BerEp4 and CK20 antibodies, as compared to that used for the one color method with peroxidase as the indicator system (with DAB as the brown chromogen) may improve these immunomarkers in dual color immunostaining.

## ACKNOWLEDGMENTS

The authors appreciate and thank Glen Dawson, BS, HT, IHC(ASCP), Jerome Jacobson, HT, QIHC(ASCP), and Katherine Wertz, BS,HTL,QIHC(ASCP) for the technical immunocytochemistry assistance. We also thank Anushree Shidham for secretarial and copy-editing support.

This study was presented in part at 96th Annual Meeting of United States and Canadian Academy of Pathology, March 24-30, 2007, San Diego, CA, USA.

## COMPETING INTEREST STATEMENT BY ALL AUTHORS

No competing interest to declare by any of the authors.

## AUTHORSHIP STATEMENT BY ALL AUTHORS

All authors of this article declare that we qualify for authorship as defined by ICMJE http://www.icmje.org/#author.

Each author has participated sufficiently in the work and take public responsibility for appropriate portions of the content of this article.

Each author acknowledges that this final version was read and approved.

## ETHICS STATEMENT BY ALL AUTHORS

This study was conducted with approval from Institutional Review Board (IRB) (or its equivalent) of all the institutions associated with this study.

Authors take responsibility to maintain relevant documentation in this respect.

## EDITORIAL/PEER-REVIEW STATEMENT

To ensure the integrity and highest quality of CytoJournal publications, the review process of this manuscript was conducted under a double blind model (authors are blinded for reviewers and reviewers are blinded for authors) through automatic online system conducted by the academic editor designated for this manuscript – **Momin Siddiqui**, MD, FIAC, Emory University Hospitals, Atlanta, GA, USA (momin.siddiqui@emoryhealthcase.org) to complete the entire peer review process stating with assignment of the reviewer to final stage of accepting /rejecting the manuscript based on reviewer input.
